# Adaptive Immunosuppression in Lung Transplant Recipients Applying Complementary Biomarkers: The Zurich Protocol

**DOI:** 10.3390/medicina59030488

**Published:** 2023-03-02

**Authors:** Macé M. Schuurmans, Miro E. Raeber, Maurice Roeder, René Hage

**Affiliations:** 1 Division of Pulmonology, University Hospital Zurich, 8091 Zurich, Switzerland; 2 Faculty of Medicine, University of Zurich, 8032 Zurich, Switzerland; 3 Department of Immunology, University Hospital Zurich, 8091 Zurich, Switzerland

**Keywords:** lung transplantation, immunosuppression, therapeutic drug monitoring, lymphocytes, eosinophils

## Abstract

Achieving adequate immunosuppression for lung transplant recipients in the first year after lung transplantation is a key challenge. Prophylaxis of allograft rejection must be balanced with the adverse events associated with immunosuppressive drugs, for example infection, renal failure, and diabetes. A triple immunosuppressive combination is standard, including a steroid, a calcineurin inhibitor, and an antiproliferative compound beginning with the highest levels of immunosuppression and a subsequent tapering of the dose, usually guided by therapeutic drug monitoring and considering clinical results, bronchoscopy sampling results, and additional biomarkers such as serum viral replication or donor-specific antibodies. Balancing the net immunosuppression level required to prevent rejection without overly increasing the risk of infection and other complications during the tapering phase is not well standardized and requires repeated assessments for dose-adjustments. In our adaptive immunosuppression approach, we additionally consider results from the white blood cell counts, in particular lymphocytes and eosinophils, as biomarkers for monitoring the level of immunosuppression and additionally use them as therapeutic targets to fine-tune the immunosuppressive strategy over time. The concept and its rationale are outlined, and areas of future research mentioned.

## 1. Introduction

Three decades ago, before the introduction of thiopurine methyltransferase (TPMT) enzyme level testing, azathioprine dosing was adjusted according to total leucocyte counts, with a cut-off at 3.5 or 3.0 G/L. This was a simple strategy using a biomarker to monitor drug effects [1,2,3]. While azathioprine was one of the commonly applied immunosuppressive drugs in the early days of lung transplantation, it has been largely replaced by mycophenolate mofetil (MMF), after the latter demonstrated a decreased incidence of biopsy-proven acute cellular rejection and chronic allograft dysfunction [4].

Lung transplantation was initiated in Zurich, Switzerland, in 1992 by the thoracic surgeon Walter Weder and the pulmonologist Rudolf Speich. In the initial phase, the program benefited from experiences obtained from the Toronto lung transplant program. The research of these early Swiss transplant specialists focused on the anastomotic technique and handling of infections in lung transplant recipients as well as the characterization of chronic rejection as “bronchiolitis obliterans syndrome”. By the turn of the century, 88 transplantations had been performed and by the end of 2022, we count 619 lung transplantations conducted in Zurich. For nearly 3 decades, ciclosporin has been our first choice of calcineurin inhibitor (CNI) except in pediatric candidates where tacrolimus is preferred. 

As in many transplant centers, therapeutic drug monitoring is the mainstay for adapting the drug dosing mainly for CNIs (namely ciclosporin and tacrolimus), and to a lesser extent for MMF and the mammalian-target of rapamycin (mTOR) inhibitor everolimus [5]. Frequently, CNI drug target levels are lowered in patients with relevant kidney dysfunction due to their nephrotoxicity and impaired immune responses due to the renal failure itself [6]. Further deterioration in kidney function should be avoided since it reduces quality of life, graft survival, and may lead to end-stage renal disease. A relevant proportion of lung transplant recipients (LTRs) requires renal replacement therapy, i.e., dialysis or even kidney transplantation. In patients with reduced kidney function, we adapt target drug levels, especially for CNIs or CNIs in combination with everolimus (Table 1).

At our center, we have also been adjusting immunosuppression by applying complementary biomarkers retrieved from differential blood counts [7,8,9]. Naturally, we have observed total leukocyte counts and total neutrophil counts, but in most cases, these do not pose a major concern unless certain antibiotic or antiviral agents with bone marrow suppressing side effects are used simultaneously, or in the elderly, where the bone marrow reserves are reduced. Nevertheless, severe neutropenia (<0.5 G/L) is associated with decreased survival and increased infection rates and should be managed by dose reduction in immunosuppressive therapy [10].

## 2. Serum Lymphocyte Counts

**Figure 1:**
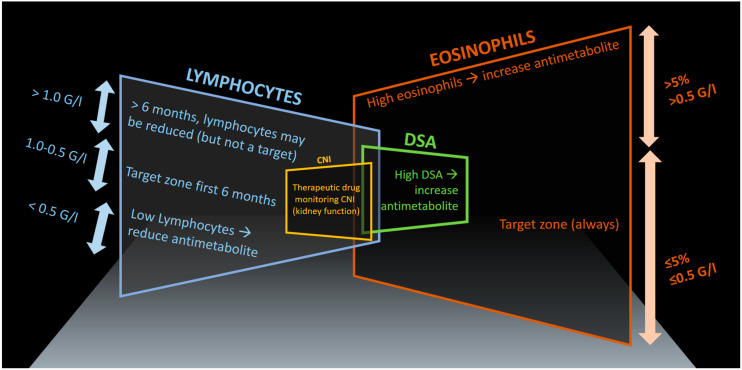
Adaptive immunosuppression considering differential white blood cell counts (eosinophils, lymphocytes) and other factors (therapeutic drug monitoring levels of calcineurin inhibitors (CNI), kidney function, and donor specific antibodies (DSA). We refer to this concept as the “Rule of Five” (the number five appearing in many of the main target values), showing graphically the components of the immunosuppressive therapy including target zones of lymphocytes and eosinophils.

Our approach mainly focuses on total lymphocyte and eosinophil counts and, if in doubt, we consider the percent values of these cells in relation to total leukocytes, thus taking a possible leukocytosis into account (Figure 1).

As a rule of thumb, we keep lymphocytes suppressed during the first six months, targeting lymphocyte counts just below 1 G/L and above 0.5 G/L. Lower lymphocyte counts will increase the risk of infections disproportionally (Figure 1). If lymphocyte counts fall below 0.5 G/L, we reduce the antimetabolite (mycophenolate) drug dosage irrespective of measured mycophenolic acid (MPA) serum drug levels. There are only very few exceptions to this rule, such as low lymphocyte counts during mild viral infections or extra corporeal photopheresis (ECP), when antimetabolite dosages are already low and the patient condition is stable without recurrent infections. In these situations, we maintain the antimetabolite dose. 

With our standard daily dose of 2 × 1.5 g mycophenolate mofetil combined with a moderate dose of prednisone (i.e., 0.5 mg/kg body weight early post-transplant) we usually achieve suppressed lymphocyte counts during the first few months post-transplant. In underweight patients, we initially apply 2 × 1 g daily. We do not increase the mycophenolate above 2 × 2 g (this is rarely used, since it is an off-label dosage in Switzerland). In our experience, the elderly have a reduced bone marrow reserve and are more sensitive to myelotoxic compounds, in particular when combinations are used such as combinations of mycophenolate, valaciclovir, and myelosuppressive antibiotics such as piperacillin/tazobactam [7]. Additionally, we take the presence of HLA antibodies and in particular donor-specific antibodies (DSA) into account during the tapering of overall immunosuppression. Our treat-to-target approach aims for the lowest possible immunosuppression preventing rejection, thus reducing organ toxicity and infections. Fearing rejection, this simple concept is often ignored by transplant physicians in our setting, favoring more intense immunosuppressive strategies, if in doubt.

## 3. Serum Eosinophil Counts

As a third biomarker, we monitor eosinophil counts and aim for values <0.5 G/L (or less than 5%) during the entire post-lung transplant follow-up (Figure 1). Often, eosinophil counts remain very low under the immunosuppressive triple therapy, with values ranging between 0 to 0.25 G/L. Once they tend to increase over time, we check for signs of rejection, the overall immunosuppression (considering the steroid dose, the CNI dose, the antimetabolite dose, signs of viral replication CMV, etc.), and finally the presence of HLA-antibodies and DSA to assess if there is any evidence of insufficient immunosuppression or, less likely in this situation, over-immunosuppression (i.e., no antibodies at all, but possibly viral replication: EBV or CMV). For the long-term management of immunosuppression, we mainly take the evolution of drug dosages, lung function, and renal function into account. Therefore, since the start of the Zurich lung transplantation program in 1992, we have recorded drug dosages, drug levels, lung function, renal function, differential blood counts, C-reactive protein, creatinine, and other biomarkers of each individual patient in a database-spreadsheet, allowing us to monitor changes over time and assisting our decision-making process on modifications to improve individualized immunosuppression. Of course, we also search for other reasons for increased eosinophils, such as primary eosinophilia and secondary causes including parasitic infections, but usually only in cases with high eosinophilia, i.e., serum eosinophils >0.5 G/L or >5%. Finally, bronchoalveolar lavage (BAL) differential blood-cell counts may also be a potential marker for allograft rejection [8,9]. The disadvantage of BAL sampling, however, is that it requires a more invasive strategy (bronchoscopy) to obtain the sample, which is more time-consuming as well as resource-intensive and poses an increased risk to the patient compared to a simple differential blood count.

## 4. Monitoring for Acute Rejection by Surveillance Bronchoscopies and Tapering Steroid Dose

We perform four to six surveillance bronchoscopies during the first year post-transplant. The obtained information from cytological (BAL) and histological samples (transbronchial biopsies, mucosal biopsies, or cryobiopsies) helps us taper or modify immunosuppression in this first post-transplant year, leading to a monthly reduction in the steroid doses by 5 mg if no signs of rejection are documented. Based on our preliminary experience, cryobiopsies tend to have a higher diagnostic yield in the evaluation of acute allograft dysfunction as compared to transbronchial biopsies [11]. In the case of acute allograft rejection (ISHLT > 2), we implement a steroid pulse therapy ranging from oral glucocorticoids (ISHLT A2) to high-dose glucocorticoids with methylprednisolone 500 mg to 1 g per day for 3 days (ISHLT A3–4). At the same time, the immunosuppressive therapy is adjusted by evaluating a CNI switch between ciclosporin and tacrolimus, or a switch between other compounds such as prednisone and prednisolone or mycophenolate mofetil and mycophenolic acid. We rarely introduce everolimus during the first 6 months after lung transplantation but consider it at a later stage, especially in the context of progressive renal failure [12]. Sometimes, we add azithromycin or pravastatin, and rarely montelukast for immunomodulation in this early phase when evidence of allograft dysfunction becomes evident. We typically perform a follow-up bronchoscopy approximately 4 to 6 weeks after modification of immunosuppression, especially when higher grades of acute allograft dysfunction were documented histologically. 

## 5. Some Practical Dosing Rules and Consideration of Renal Dysfunction

A short word on immunosuppression medication dosing principles. We usually try to aim for symmetric doses, i.e., the same dose of CNI or antimetabolite in the morning and evening. However, when fine-tuning is necessary and specific low-dosed tablets are not provided by the pharmaceutical industry to allow precise dose adjustments, it is not always possible to stick to this principle and so we no longer aim for symmetry. If the CNI dose is asymmetric, the higher dose is given in the morning, due to the higher water/liquid intake during the day. If the antimetabolite dose is asymmetric, the higher dose is prescribed in the evening in order to counterbalance the morning-only dose of steroids and a higher morning dose of CNI (in the case of asymmetric dosing). With the advent of extended-release tacrolimus, we quite often give tacrolimus only in the morning and fine-tune the immunosuppression or the tacrolimus levels by altering the co-medication, in particular the itraconazole dosing. As a rule of thumb, we give itraconazole life-long and concomitantly with the CNI, thus benefiting from the drug metabolism inhibition of this agent, leading to substantially lower drug dosing requirements for the CNI drugs. This is a major cost-saving factor using a low-cost antifungal agent, thus also preventing some typical fungal infections. In the dosing of itraconazole, we usually start with 200 mg twice daily and adjust the dose according to drug levels, aiming for levels of 0.5–1.0 mg/L. An electrocardiogram is routinely performed when measurable itraconazole levels have been documented, usually about 3–5 weeks post-transplant, and again once maintenance immunosuppression has been established (i.e., at one-year post-transplant) in order to detect a possible prolonged QT time. 

In the long-term management of LTR with deteriorating kidney function, we consider the combination of everolimus with low-dose tacrolimus to limit further deterioration of renal function. In this combination, the target drug levels are 3–5 μg/L for both drugs. Sometimes, we even reduce these target ranges further to 2–4 μg/L for both drugs in cases of severe renal failure (eGFR < 30).

## 6. Extracorporeal Photopheresis for Chronic Lung Allograft Dysfunction

A further strategy to modify immunosuppression is to introduce extracorporeal photopheresis (ECP). This additional method of influencing and modifying the immune response allows us to reduce the dose of the pharmacological immunosuppressive agents, in particular the CNI in the case of nephrotoxicity or more uncommon CNI-adverse effects, such as posterior reversible encephalopathy syndrome (PRES). In case of malignancies such as skin cancer or post-transplant lymphoproliferative disorder (PTLD), the introduction of ECP allows a reduction in the antimetabolite dosing. Due to cost regulations in Switzerland, the patient must also have been diagnosed with chronic lung allograft dysfunction (CLAD) and all known immunomodulatory strategies should have been applied. In cases of cutaneous carcinogenesis, affecting approximately 20% of our LTRs, mTOR inhibitors (everolimus) are preferred instead of an antimetabolite [13]. It is noteworthy that everolimus can significantly delay wound-healing. Therefore, we stop everolimus 1–2 weeks prior to elective surgery and restart it after complete wound-healing. In rare cases, everolimus is used to replace the CNI completely (i.e., cancer diagnosis with chemotherapy). 

## 7. Why Consider Eosinophils and Lymphocytes?

The exact relationship of eosinophil counts and allograft rejection is not fully understood and many investigators are still trying to dissect the complex interaction of key mediators in transplant alloimmunity [14,15,16,17,18,19,20,21]. For several years, we have considered eosinophils as an epiphenomenon highly correlating with lung allograft rejection, which is supported by recent research [18]. A key cytokine in eosinophil homeostasis is interleukin (IL)-5, which is secreted by type 2 helper (Th2) T cells and the more recently identified type 2 innate lymphoid cells (ILC2) [22,23]. The latter are tissue-resident cells expressing the IL-33 receptor mediating type-2 immune responses including parasite clearance and allergic responses, especially in the lungs [24,25]. It is thus interesting to hypothesize that increased eosinophil counts are either a biomarker for a Th2-mediated graft rejection and/or damaged lung graft epithelial cells, which by secreting IL-33 promote IL-5-expressing ILC2s, eventually inducing eosinophilia. However, the precise mechanism of lung allograft rejection, and especially the sometimes contradictory roles of helper T-cell subsets, is incompletely understood [26]. Due to the emergence of biologics targeting the Th2 axis by blocking IL-4 and IL-13 (e.g., with dupilumab) or IL-5 signaling (e.g., with mepolizumab and benralizumab), understanding the mechanism of eosinophilia in the lung transplant setting is of great importance to act as the basis for bringing these drugs into use in novel indications such as chronic allograft dysfunction.

However, we have considered eosinophil counts as a supporting biomarker when modifying overall immunosuppression. We have been doing this due to a lack of other widely available biomarkers, which could represent the level of overall immunosuppression. Not only have we closely observed eosinophil counts in the post-transplant setting, but we have aimed to keep them in a target zone between ≤0.5 G/L and ≤5%, respectively (Figure 1). In case of increased values, both the corticosteroids and the antimetabolite mycophenolate are most effective in reducing eosinophil counts. Since increasing the corticosteroid dose has its disadvantages due to known adverse effects, our main focus has been on increasing the mycophenolate in stable patients without signs of acute allograft dysfunction, defined by lung function decline. In acute allograft dysfunction, we apply steroid pulses as described above. In all other cases, a slight increase in mycophenolate dose is usually sufficient to reverse the trend of increasing eosinophil counts. An analogue phenomenon that we have observed is with DSA, which we attempt to detect after 3, 6, and 12 months and subsequently every 12 months unless a previous sample has shown DSAs. In that case, we measure DSA levels every 3 months, particularly when we have adjusted the immunosuppression, generally by increasing the antimetabolite. We usually increase the daily mycophenolate dose by 250 mg or 500 mg mycophenolate mofetil or 180 mg or 360 mg of mycophenolic acid for the enteric-coated preparation. As a rule, we rarely use serum MPA values to guide dosing mainly because there is insufficient evidence that therapeutic drug monitoring for this component improves lung transplant outcomes. If we measure mycophenolate levels, it is usually to assess drug adherence and intestinal absorption, as sometimes bioavailability can be an issue.

## 8. Over-Immunosuppression and Calcineurin Inhibitor Therapeutic Drug Monitoring

Over-immunosuppression also has drawbacks including infections (CMV, EBV, CARV), carcinogenesis, and PTLD. Therefore, the most appropriate target values for eosinophils as a biomarker for overall immunosuppression remain to be determined, which may also include a lower limit as part of the target range. In the future, there may be better measures, such as cell-free DNA or Torque teno virus, for estimating overall immunosuppression, which would guide us better in tailoring immunosuppressive drug doses. However, in the meantime, we rely on the “crude” value of eosinophils and lymphocytes.

The target levels of CNI are tailored individually depending first on the documented acute cellular rejection episodes, presence of CLAD, and quantitative counts of CMV and EBV, and secondly on CNI side effects, the presence of kidney dysfunction, and the presence of DSA. We also measure total IgG values and substitute them by intravenous immunoglobulins in cases of recurrent infections and a total IgG level below the reference value. 

Therapeutic drug monitoring of ciclosporin after lung transplantation has traditionally used trough (C0) levels [27]. However, C0 levels have a poor correlation with area-under-the-curve (AUC) measurements of ciclosporin exposure [28]. Hence, in patients with ciclosporin, the area under the curve (AUC) is determined after 3 months post-transplantation, then after 6 months and annually thereafter. This helps us to determine if the Cmax is after one or (more often) 2 h following drug intake (C1 or C2-type), and allows us to determine not only trough levels but also Cmax-values, and to calculate the AUC for the 4 h, giving us an idea of drug exposure. Cmax values are supposed to correlate much better with the ciclosporin exposure measured by AUC. In addition, using C1/C2 as target values might be associated with reduced rates of acute cellular rejection and CLAD of the bronchiolitis obliterans phenotype. Table 1 shows our target levels for CNIs, which have been compiled from various sources and slightly adapted to our clinical context, which includes an induction treatment with basiliximab after transplantation. Age above 60 years has been considered as a target-dose lowering-factor, since our long-standing experience has shown that advanced age can be more safely treated with CNI drug target levels one step lower than for younger LTRs. Our approach to immunosuppression is in many ways similar to strategies by other institutions, but the personalized medicine approach adapts the immunosuppressive strategy to the lymphocyte and eosinophil counts. Studies are required to delineate if this strategy is associated with better outcomes, what underlying mechanisms may predominantly define lymphocyte and eosinophil counts, and if this strategy can compete with newer strategies that use other markers for the level of overall immunosuppression, such as cell-free DNA or Torque teno virus-based approaches. 

In conclusion, adaptive immunosuppression for lung transplant recipients incorporates many known principles of the triple immunosuppressive treatment and additionally considers the white blood cell counts, in particular lymphocyte and eosinophil counts. These values are considered a proxy for the level of immunosuppression and the underlying cytokine patterns and can be correlated with allograft dysfunction. Certain target values are considered in a time-dependent manner as displayed in Figure 1, which captures key elements considered in the personalized approach to immunosuppression. This concept needs to be studied in different settings and compared with newer methods of quantifying and guiding overall immunosuppressive levels by measuring donor-derived cell-free DNA and Torque teno virus levels. The advantage of this approach is that it uses highly standardized and widely available laboratory measurements. 

## Figures and Tables

**Table 1:** Therapeutic drug monitoring target values.

Target Ciclosporin (Sandimmun) C0 Levels (Trough Levels)		
Time post-transplant	eGFR ≥ 50	eGFR < 50
week 1	200–300	150–200
up to 3 months	250–300 **	180–200 **
3–6 months	150–250	100–180
6–12 months	120–200/AUC	80–160/AUC
12–24 months	90–180/AUC	70–140/AUC
>24 months	Individual target (AUC)	Individual (AUC)
Target Ciclosporin (Sandimmun) C2 levels (Cmax, sometimes C1)		
Time post-transplant	eGFR ≥ 50	eGFR < 50
week 1	1100–1300	700–800
up to 3 months	1000–1200 **	750–800 **
3–6 months	900–1100/AUC	500–700/AUC
6–12 months	700–900/AUC	400–600/AUC
12–24 months	500–700/AUC	300–500/AUC
>24 months	Individual target (AUC)	Individual (AUC)
Target Tacrolimus (Prograf, Advagraf) trough levels, mg/L		
Time post-transplant	eGFR ≥ 50	eGFR < 50
week 1	11–15	8–11
up to 3 months	10–12 **	8–10 **
3–6 months	9–11	7–9
6–12 months	8–10	6–8 ***
12–24 months	7–9 ***	5–7 ***
>24 months	6–8 ***	4–6 ***

MMF/MPA trough levels: 2–5 mg/mL (as orientation; mainly controlled by differential blood counts). AUC = area under the curve; MMF = mycophenolate mofetil (CellCept); MPA = mycophenolic acid (Myfortic); Everolimus trough level: 3–5 ng/mL. Note: Everolimus is avoided in the first 1–2 months after transplantation due to antifibrotic activity and potential wound healing problems. ** if patient is 60 years or older, then the target range is one level lower (applicable to the whole timeframe). *** if everolimus is given, then 3–5 mg/L target level for both everolimus and tacrolimus (beyond 9 months post-transplant).

## Data Availability

Not applicable.
